# Characterization of Genetic Variants in the SLC5A5 Gene and Associations With Breast Milk Iodine Concentration in Lactating Women of African Descent: The NUPED Study

**DOI:** 10.3389/fnut.2021.692504

**Published:** 2021-07-23

**Authors:** Sicelosethu S. Siro, Jeannine Baumgartner, Maryke Schoonen, Jennifer Ngounda, Linda Malan, Elizabeth A. Symington, Cornelius M. Smuts, Lizelle Zandberg

**Affiliations:** ^1^Centre of Excellence for Nutrition, North-West University, Potchefstroom, South Africa; ^2^Human Nutrition Laboratory, Department of Health Sciences and Technology, Institute of Food, Nutrition and Health, ETH Zurich, Zurich, Switzerland; ^3^Department of Nutrition and Dietetics, University of the Free State, Bloemfontein, South Africa; ^4^Department of Life and Consumer Sciences, University of South Africa, Johannesburg, South Africa

**Keywords:** breast milk, iodine concentration, lactating women, *SLC5A5* gene, sodium iodide symporter, urinary iodine concentration

## Abstract

**Background:** The sodium iodide symporter is responsible for the transfer of iodine into breast milk and is encoded for by the *SLC5A5* gene. The role of genetic variants in the *SLC5A5* gene locus in relation to the transfer of iodine from plasma into breast milk in healthy lactating individuals has, to our knowledge, not been explored.

**Objective:** To identify and characterize possible genetic variants of the SLC5A5 gene in women of African descent living in urban South Africa, and to study associations with breast milk iodine concentrations (BMIC) in lactating women.

**Methods:** This study is affiliated to the Nutrition during Pregnancy and Early Development (NuPED) cohort study (*n* = 250 enrolled pregnant women). In a randomly selected sub-sample of 32 women, the *SLC5A5* gene was sequenced to identify known and novel variants. Of the identified variants, genotyping of selected variants was performed in all pregnant women who gave consent for genetic analyses (*n* = 246), to determine the frequency of the variants in the study sample. Urinary iodine concentration (UIC) in spot urine samples and BMIC were measured to determine iodine status. Associations of SLC5A5 genetic variants with BMIC were studied in lactating women (*n* = 55).

**Results:** We identified 27 variants from sequencing of gene exomes and 10 variants were selected for further study. There was a significant difference in BMIC between the genotypes of the rs775249401 variant (*P* = 0.042), with the homozygous GG group having lower BMIC [86.8 (54.9–167.9) μg/L] compared to the (A) allele carriers rs775249401_(AG+AA)_ [143.9 (122.4–169.3) μg/L] (*P* = 0.042). Of the rs775249401_(GG)_, 49% had UIC <100 μg/L and 61% had BMIC <100 μg/L. On the other hand, 60% of the rs775249401_(AG+AA)_ carriers had UIC <100 μg/L, and none had a BMIC <100 μg/L.

**Conclusion:** Our results suggest that A-allele carriers of rs775249401_(AG+AA)_ are likely to have higher iodine transfer into breast milk compared to the homozygous GG counterparts. Thus, genetic variations in the SLC5A5 gene may play an important role in the transfer of iodine from plasma into breast milk and may partially explain inter-individual variability in BMIC.

## Introduction

The sodium iodide symporter (NIS) is an intrinsic plasma membrane glycoprotein mediating iodide uptake into thyroid follicular cells. The NIS protein consisting of 643 amino acids is encoded by the *SLC5A5* gene, which is located on the forward strand of chromosome 19 (19: 17,982,754–18,005,983, GRCh37.p12) with an open reading frame consisting of 1929 nucleotides arranged as 14 introns and 15 exons (1). The NIS plays a crucial role in iodine metabolism and thyroid regulation (2). Besides thyroid hormone synthesis, the NIS is expressed in breast tissue during late pregnancy and lactation and is responsible for the transfer of iodine from plasma into mammary epithelial cells of lactating breasts (3).

Breast milk is a crucial source of iodine for the breastfeeding infant (4). Thus, adequate breast milk iodine concentration (BMIC) is important for meeting the iodine requirements of infants. Maternal iodine intake, estimated by measuring urinary iodine concentration (UIC), is known to greatly influence BMIC. Therefore, lactating women with insufficient iodine intake reportedly excrete insufficient breast milk iodine to meet the infants' needs (4–6). BMIC of 92 to 150 μg/L have been suggested to provide sufficient iodine to infants (6–8), but consensus on a BMIC threshold to define adequate iodine nutrition has not yet been reached. However, mean and median BMIC values were shown to vary widely between areas, with values typically ranging from <50 μg/L in iodine-deficient areas (4) to 100–150 μg/L in areas of iodine sufficiency (7) but being as high as 150–180 μg/L in areas of adequate iodine supply (9, 10). In a previous cross-sectional study in a convenience sample of 100 lactating women living in a semi-urban area of South Africa, we observed a median BMIC of 179 (126–269) μg/L with large inter-individual variations (11).

Previous research has shown that there is a preferential fractional excretion of iodine in breast milk rather than urine among participants with poor iodine status living in iodine sufficient regions (12). Additionally, women from iodine-deficient regions showed a constant partitioning of iodine into breastmilk. As such, participants with suboptimal iodine status have been shown to present with adequate BMIC. This could be explained by a protective mechanism that allows for a steady supply of iodine to breastfed infants of lactating women with suboptimal iodine status (12, 13), and the NIS is likely to play a major role.

Since there are limited data available on the role of genetic variants in the *SLC5A5* gene in relation to breast milk iodine concentration, we aimed to characterize genetic variants in the *SLC5A5* gene of women of African descent living in urban South Africa. Further, we investigated the relationship between selected variants with breast milk iodine concentration in lactating women.

## Materials and Methods

### Study Design and Site

The NuPED study was a prospective cohort study conducted in Johannesburg, South Africa from March 2016 to July 2018. The study protocol has be previously published (14). In brief, pregnant women (*n* = 250) were enrolled if they were between 18 and 39 years of age, <18 weeks gestational age, born in South Africa or a neighboring country, have lived in Johannesburg for at least 12 months, were able to communicate effectively in one of the local languages, non-smoking, and expecting a singleton. Pregnant women were excluded from participation if they reported use of illicit drugs, had a known non-communicable disease such as diabetes, renal disease, history of high blood cholesterol and hypertension, and had a known infectious disease such as tuberculosis or hepatitis, or known serious illness such as cancer, lupus or psychosis. HIV positive women were included in the study. Pregnant women were assessed at <18, 22, and 36 weeks gestation. Follow-up assessments in the women and their infants were performed at 6, 7.5, and 12 months after birth. Of the 250 enrolled women, a total of 98 mother-infant pairs participated in the 6-month follow-up.

In a randomly selected sub-sample of 32 women, the *SLC5A5* gene was sequenced to identify and characterize genetic variants. Of the identified variants, genotyping of selected variants was performed in all pregnant women enrolled in the NuPED study and who gave consent for genetic analyses (*n* = 246), to determine the frequency of the variants in the study sample.

This study was conducted according to the guidelines of the Declaration of Helsinki and all procedures involving human participants were approved by the Human Research Ethics Committees (HREC) of the North-West University (NWU-00186-15-A1 for the NuPED pregnancy phase, NWU-00049-16-A1 for the postnatal phase) and the University of the Witwatersrand, Johannesburg (M150968 and M161045). These studies were also reviewed by the Rahima Moosa Mother and Child Hospital (RMMCH) research review committee, the Gauteng Department of Health, and the Johannesburg Health District's District Research Committee. Further ethical approval was granted for this sub-study by the North-West University HREC (NWU-00455-19-S1 for this study). All participants gave written informed consent.

### Blood Sample Collection and Genetic Analysis

#### Genomic DNA Isolation and Next-Generation Sequencing

A venous blood sample was collected into trace element-free ethylenediaminetetraacetic acid (EDTA)-coated vacutainer tubes via venepuncture of the antecubital area of the arm. Blood samples were processed by centrifugation (at 2,000 rpm for 15 min) within 1 h after blood collection to separate plasma, red blood cells and buffy coat. The buffy coat was aliquoted and stored in a 1:1 vol: vol RNA*later* (Ambion, Thermo Fischer Scientific) on-site at −20°C for a maximum of seven days. The samples were then transported on dry ice to the Centre of Excellence for Nutrition (CEN) laboratories in Potchefstroom, South Africa and stored at −80°C for genomic DNA (gDNA) isolation.

Genomic DNA (gDNA) was isolated from buffy coat using the Maxwell® 16 instrument and Maxwell® 16 DNA Purification Kit (AS1010) (Promega Corporation) following the manufacturer's instructions. Quantification of gDNA was done using the NanoDrop® ND-1000 UV-Vis Spectrophotometer (Thermo Fischer Scientific).

Massively parallel next-generation sequencing (NGS) was performed using the Ion Torrent platform on the subset of 32 randomly selected samples. A custom Ion Ampliseq panel was designed with the online Ampliseq designer (https://ampliseq.com/, last accessed July 2020). This customized panel included the *SLC5A5* gene locus spanning a target region size of 20.83 kbp with 20 amplicons covering 100% of the targeted sequence. Library preparation was performed on the Ion Chef® as per the manufactures specifications. A total of 10 ng gDNA (0.67 ng/μl) was used as input volume for library preparation. Library and template preparation were done with the Ion AmpliSeq™ Kit for Chef DL8 for 32 reactions (Cat number: A29024,) and the Ion 510™ and Ion 520™ and Ion 530™ Kit—Chef (Cat number A34019), respectively. The entire coding region of each selected gene, including flanking regions of introns-exons was sequenced according to 200bp chemistry, using an Ion 530™ Chip Kit-4 Reactions (Cat number: A27763) and the Ion S5™ System (Cat number: A27212, ThermoFisher, MA, USA).

Data analysis of raw reads obtained from the Ion S5™ System was done with the Torrent Suite (v.5.8). The fastq files were uploaded to the sequence reads archive Bioproject PRJNA735618. The primary analysis included signal calling and base-calling. Quality control of the bases was filtered according to a Phred quality score of 20, depth > 10 and quality score of >500 frets were met. The sequence files were aligned against Genome Reference Consortium Human Build 37 (hg19), followed by coverage analysis and variant calling using the coverage analysis and variantCaller plugins from the Torrent Suite, respectively. Secondary data analyses of the variant caller files were annotated, filtered and mined following an in-house pipeline (15).

#### Variant Selection, Genotyping, and Quality Assessment

In the subset of 32 sequenced samples, variants that passed the quality control assessment were considered for validation in the entire sample set using the iPLEX® MassARRAY system from Agena Bioscience™. IPLEX assays were designed and analyses were performed by the service provider Inqaba Biotech (Inqaba Biotechnology Pretoria South Africa). Assays were designed using the Assay Design Suite (ADS) software and dbSNP for metadata. gDNA was amplified in 96 microtiter plates using iPLEX reagent kits and a nano dispenser RS1000 was used to transfer samples from microtiter plates to a SpectroCHIP® array. Data were obtained from the SpectroCHIP® array using the MassARRAY® analyser. Reports were automatically generated by Typer (Company). Genotype calls were made in real-time during MALDI-TOF analysis and data was automatically saved to the MassARRAY database. Variants were assessed for quality, and tested for adherence to Hardy-Weinberg equilibrium (HWE) (16) by using Haploview and modified Pearson chi-square (χ^2^) test. Adherence to HWE was set at *P* < 0.001.

### Urine Sample Collection and Iodine Analysis

From the 98 women participating in the 6-month follow-up, we collected a midstream spot urine sample (10–40 ml) into clean polystyrene cups between 07:00 and 12:00 noon, and approximately 5 ml was decanted into iodine-free screw-capped cups. The research team ensured that the urine samples were not used for any routine assessments using dipsticks (potential contamination with iodine). Samples were aliquoted and stored on-site at −20°C for a maximum of 7 days. Thereafter, samples were transported on dry ice to the CEN laboratories, for storage at −80°C until analysis.

Urinary iodine concentration (UIC) in spot urine samples was measured in duplicate using the Pino modification of the Sandell-Kolthoff reaction with spectrophotometric detection at CEN laboratories (17, 18). All analyses were done using nanopure grade water and all laboratory glassware and plasticware were acid washed before use. Internal and external controls were used to ensure the quality of the analysis. Iodine concentrations in spot urine samples are expressed as median concentrations (μg/L). A UIC cut-off of <100 μg/L was used to indicate insufficient iodine intake in lactating women (19).

### Breast Milk Sample Collection and Iodine Analysis

From 58 lactating women who participated in the 6-month follow-up, a breast milk (foremilk) sample (≈5 ml) was collected by manual expression into an iodine-free screw-capped cup before feeding the infant. Iodine concentrations in breast milk (in μg/L) were measured using a multi-collector inductively coupled plasma mass spectrometer [MC-ICP-MS (Finnigan NEPTUNE, Thermo Scientific™ Waltham, MA, USA)] as described by Dold et al. (10). A BMIC cut-off of <100 μg/L was used to indicate inadequate maternal iodine intake (20).

### Iodine Excretion Calculations

Individual UIC and BMIC measures were used to calculate the estimated daily iodine excretion through the urine and breast milk by assuming a total daily urine volume of 1.5 L (12, 21, 22) and breast milk volume of 0.78 L (12, 22). Estimated total daily iodine excretion was calculated as the iodine excretion in urine added to the iodine excretion in breast milk (12). Furthermore, fractional iodine excretion in urine and breast milk as percentages of estimated total daily iodine excretion were also calculated (12, 22).

### Statistical Analyses

Raw data were captured in Microsoft Access and 20% of all data were randomly checked for correctness. All genetic, UIC and BMIC, data were captured in Excel Windows XP (Microsoft, Seattle, WA, USA). Data processing and statistical analysis of data were performed using SPSS software (SPSS Inc, Chicago, IL, USA).

Data were tested for normality using the Kolmogorov-Smirnov test. UIC and BMIC data were log-transformed for further analysis. For UIC, values above the cut-off indicative of excessive iodine intake (UIC >500 μg/L) were considered outliers. These UIC outliers (n = 2) were excluded from the analysis because high intakes of iodine have previously been reported to lead to improved BMIC in an individual that harbored a *SLC5A5* variant associated with the lower transfer of iodine into breast milk (23). Normally and non-normally distributed data are expressed as means ± standard deviation (SD) and medians (25^th^ percentile, 75^th^ percentile), respectively. Categorical data are expressed as frequencies and percentages.

Participants were stratified according to UIC categories (UIC <100 μg/L and UIC ≥100 μg/L) or BMIC categories (BMIC <100 μg/L and BMIC ≥100 μg/L). The between-group analyses were performed using the Mann Whitney U test. Overlaid scatterplots were used to depict the relationship between total daily iodine excretion, fractional iodine excretion in breast milk and fractional iodine excretion in urine. Unadjusted general linear models were performed to compare UIC and BMIC between genetic variants with the recessive genetic model (GG vs. GA + AA) as categorical variables. For the significant models, effect sizes were calculated using Cohens' *d* and partial eta squared. Significance was set at *p* < 0.05.

## Results

### Characterization of the Genetic Variants in the SLC5A5 Gene Locus

Genomic DNA was isolated from 246 samples and the *SLC5A5* gene of 32 randomly selected samples were sequenced following a targeted gene sequencing approach. Variants were quality controlled and 27 genetic variations passed the quality control assessment. Of the 27 variants, 26 had known annotation and one was novel located in the 5' untranslated region (UTR). Of the annotated variants, six were coding and 13 were in intronic regions, whereas the remaining seven variants were located in the 5' UTR (Table 1). Variants were inspected for possible functionality based on genomic position as well as association with regulatory sites and or available literature. Ten variants (rs121909177_(C/T)_, rs4808708_(G/A)_, rs7255301_(G/A)_, rs73520743_(A/C)_, rs112076606_(A/G)_, rs73520745_(G/A)_, rs775249401_(G/A)_, rs34850953_(G/T)_, rs8103545_(C/T)_ and the novel variant) were validated in all 246 samples (see Table 2). All variants adhered to Hardy-Weinberg Equilibrium (HWE). The minor allele frequencies (MAF) of the variants studied in the lactating women (*n* = 55) compared well with the MAF of the total group. In the total group of pregnant women (*n* = 246), none of the women were homozygous for the alternative alleles for rs121909177_(C/T)_, rs73520745_(G/A)_, rs73520743_(A/C)_, and rs8103545_(C/T)._ In addition, the lactating women had no homozygosity for the alternative alleles of rs7255301_(G/A)_ and rs34850953_(G/T)_ variants too. The total study sample (*n* = 246) was monomorphic for the CC genotype of the novel variant (19: 17983034) and the lactating women all harbored the CC genotype for rs121909177_(C/T)_ (Table 2).

**Table 1:** Genetic variants identified with next generation sequencing.

	**Reference ID**	**Location bp^**a**^**	**Reference allele^**a**^**	**Alternative allele^**a**^**	**Exon^**a**^**	**Variant type**	**Minor allele frequency (MAF)**
**Annotated variants**							
1	rs371875541	17983314	G	T	1/15^b^	synonymous	0.031
2	rs775249401	17985454	G	A		intron	0.125
3	rs55910163	17994635	C	T		intron	0.188
4	rs866765676	17982992	C	T	1/15	5′ UTR	0.031
5	rs73518702	17983105	C	A	1/15	5′ UTR	0.188
6	rs575147988	17983038	C	T	1/15	5′ UTR	0.063
7	rs34850953	17983503	G	T		intron	0.125
8	rs150814476	17988717	C	G		intron	0.125
9	rs116154266	17983093	C	T	1/15	5 prime UTR	0.063
10	rs8103545	17986763	C	T	5/15	Splice site	0.188
11	rs35209536	17988794	C	T	7/15	synonymous	0.188
12	rs73520743	17994573	A	C	11/15	synonymous	0.313
13	rs112076606	17994594	A	G		intron	0.281
14	rs73520745	17994836	G	A	12/15	missense	0.188
15	rs4808708	18001686	G	A		intron	0.281
16	rs4808709	18001839	A	G		intron	0.438
17	rs112077649	17983075	C	T	1/15	5′ UTR	0.094
18	rs77947605	17994644	C	T		intron	0.188
19	rs118133504	17983059	C	T	1/15	5′ UTR	0.031
20	rs113740966	17985371	AT	A		intron	0.031
21	rs369522814	17994641	C	G		intron	0.063
22	rs121909177	17999206	C	T	13/15	synonymous	0.031
23	rs7255301	17984834	G	A		intron	0.313
24	rs200868271	17994619	A	C		intron	0.031
25	rs8108188	17988825	T	G	7/15	missense	0.031
26	rs190567881	17988721	C	G		intron	0.031
**Novel variants**							
1		17983034	C	T^c^	1/15	5′ UTR	0.188
2		17999293	G	C^c^		intron	0.031
3		17999295	T	A^c^		intron	0.031

a *Source: Ensembl release 96—April 2019, retrieved 03 April 2019 (24)*,

b *Exon number in which the single nucleotide is found out of the 15 exons of the SLC5A5 gene*.

c *Alternative allele as observed in our population*.

**Table 2:** Molecular characteristics of SLC5A5 genetic variants among pregnant women (*n* = 264) and the subset of lactating women (*n* = 55).

				**All women (*****n*** **= 246)**					**Sample of lactating women (*****n*** **= 55)**				
	**Location**	**Gene structure^**a**^**	**Allele^**a**^**	**HWE**	**MAF**	**Genotype (*****n*****)**			**HWE**	**MAF**	**Genotype (n)**		
	**Chr:bp^**a**^**		**(M):(m)**	***P*-value**		**MM**	**Mm**	**mm**	***P*-value**		**MM**	**Mm**	**mm**
rs121909177	19:17888397	Exon	C:T	1.0	0.007	225	3	-	1.0	0	52	-	-
rs4808708	19:17890877	Intron	G:A	1.0	0.137	176	56	4	1.0	0.087	41	11	1
rs7255301	19:17874025	Intron	G:A	0.0023	0.194	126	76	1	0.2802	0.188	30	14	-
rs73520743	19:17883764	Exon	A:C	1.0	0.02	-	9	223	1.0	0.03	-	44	6
rs112076606	19:17883785	Intron	A:G	0.448	0.214	2	55	81	1.0	0.214	1	16	17
rs73520745	19:17884027	Exon	G:A	1.0	0.046	217	22	-	1.0	0.038	46	5	-
rs775249401	19:17874645	Intron	G:A	1.0	0.056	217	25	1	1.0	0.028	43	9	1
rs34850953	19:17872694	Intron	G:T	0.7938	0.077	202	33	2	1.0	0.038	45	7	-
rs8103545	19:17875954	Exon	C:T	1.0	0.05	217	24	-	1.0	0.087	46	7	-
Novel	19:17983034	5′ prime UTR	C:C	1.0	0.0	218	-	-	1.0	0	47	-	-

*Chr, chromosome; bp, base pair; M, reference allele; m, alternative; HWE, Hardy-Weinberg equilibrium; MAF, minor allele frequency*.

a *Ensembl release 96—April 2019, retrieved 03 April 2019 (24)*.

### Participant Characteristics

A total of 246 women from the NuPED study consented to participate in the genetic study of which 98 mothers and their infants were assessed at 6-months postpartum. Of these, 58 women indicated to breastfeed their infants and provided a breastmilk sample. Characteristics of the women who were lactating (*n* = 58) and non-lactating (*n* = 40) at 6 months postpartum are given in Table 3. The two groups were similar in age, height, weight, BMI, MUAC and UIC (Table 3). The median UIC of all women (*n* = 98) was 104.7 (66.2–154.0) μg/L, whereas the lactating and non-lactating women had a median UIC of 95 (64–146) μg/L and 122 (73–207) μg/L, respectively (*P* = 0.259). The lactating women had a median BMIC of 99.4 (60.1–167.9) μg/L. Of the lactating women, 53% had a UIC <100 μg/L and 51% had a median BMIC <100 μg/L. Lactating women had a total estimated iodine excretion of 227.2 (167.4–346.7) μg/d, with 36% iodine excreted in breast milk and 64% in urine.

**Table 3:** Participant characteristics.

**Characteristics**	**All (*n* = 98)**	**Lactating women (*n* = 58)**	**Non-lactating women (*n* = 40)**	^*^P-value
Age (years)	27.2 ± 4.8^α^	28.1 ± 4.7	27.9 ± 5.0	0.750
Weight (kg)	69.4 (60.1–81.6)^β^	70.4 (59.4–81.5)	68.6 (61.2–79.7)	0.702
Height (m)	159.1 ± 6.3	159.4 ± 6.3	158.4 ± 5.7	0.634
MUAC (cm)	29.8 (27.0–33.5)	30.0 (27.0–33.6)	29.0 (27.0–33.5)	0.757
BMI (kg/m^2^)	27.5 (24.3–32.4)	28.1 (24.1–32.4)	26.2 (24.8–32.3)	0.831
**Country of birth (*****n*** **= 95) [*****n*****(%)]**				
South Africa	68 (69.4)	36 (64.3)	29 (82.9)	0.044
Zimbabwe	26 (26.5)	20 (35.7)	5 (14.3)	
Lesotho	1 (1.0)	–	1 (2.9)	
**Years in South Africa (*****n*** **= 95) [*****n*****(%)]**				
1–2 years	3 (3.1)	3 (5.4)	–	0.183
>2–5 years	6 (6.1)	5 (8.9)	1 (2.9)	
>5 years	86 (87.8)	48 (85.7)	34 (97.1)	
**Ethnicity (*****n*** **= 97) [*****n*****(%)]**				
Black	86 (88.6)	52 (89.7)	31 (88.6)	0.676
Colored	10 (10.2)	5 (8.6)	4 (11.4)	
Indian	1 (1.0)	1 (1.7)		
**Education (*****n*** **= 97) [*****n*****(%)]**				
None/primary	4 (4.1)	2 (3.4)	2 (5.7)	0.324
Grade 8-10	14 (14.3)	7 (12.1)	6 (17.1)	
Grade 11-12	55 (56.1)	31 (53.4)	22 (62.9)	
Tertiary	24 (24.5)	18 (31.0)	5 (14.3)	
**Employment (*****n*** **= 97) [*****n*****(%)]**				
Unemployed	44 (44.9)	28 (48.3)	15 (42.9)	0.640
Self-employed	5 (5.1)	3 (5.2)	2 (5.7)	
Wage earner	46 (46.9)	25 (43.1)	18 (51.4)	
Other	2 (2.0)	2 (3.4)	–	
**Marital status (*****n*** **= 97) [*****n*****(%)]**				
With partner	64 (66.0)	40 (69.0)	21 (60.0)	0.378
Without partner	33 (34.0)	18 (31.0)	14 (40.0)	
**Grants (*****n*** **= 92) [*****n*****(%)]**				
None	65 (66.3)	44 (81.5)	19 (55.9)	0.061
Child support	24 (24.5)	10 (18.5)	12 (35.3)	
Social relief	1 (1.0)	–	1 (1.0)	
Disability	1(1.0)	–	1 (1.0)	
Old age	1 (1.0)	–	1(1.0)	
**LSM score [*****n*****(%)]**				
1–4 (Low)	3 (3.0)	2 (3.4)	1 (2.8)	0.250
5–7 (Medium)	62 (63.3)	32 (55.2)	26 (72.2)	
8–10 (High)	33 (33.7)	24 (41.4)	9 (25.0)	
**HIV status**				
HIV-infected [*n* (%)]	28 (28.6)	16 (44.4)	11 (19.0)	0.008
**Median UIC (μg/L)**	105 (66–154)	95 (64–145.5)	122 (73–207)	0.259
**Median BMIC (μg/L)**	–	104 (60–169)	–	
**Estimated daily iodine excretion (μg/d)**				
Urine^γ^	157.0 (99.3–231.0)	142.4 (96.5–218.3)	178.4 (168.8–309.5)	0.259
Breast milk^δ^	–	80.9 (46.8–132.1)	–	
Total daily estimated daily iodine excretion^ε^	–	227.2 (167.4–346.7)	–	

*MUAC, mid-upper arm circumference; BMI, body mass index; LSM, living standards measure; UIC, urinary iodine concentration; BMIC, breast milk iodine concentration*,

α *Mean ± SD (all such values)*,

β *Median; 25th and 75th percentiles in parenthesis (all such values)*,

γ *Individual estimated iodine daily excretion in urine was calculated by multiplying UIC by an assumed total daily urine volume of 1.5 L (12, 21, 22)*,

δ *Individual estimated iodine daily excretion in breast milk was calculated by multiplying individual BMIC by an assumed total daily breast milk volume of 0.78 L (12, 22)*.

ε *Estimated total daily iodine excretion is the sum of iodine excretion in urine and iodine excretion in breast milk (12)*.

* *p-value for the Mann Whitney U test significance set at p ≤ 0.05*.

### Associations of SLC5A5 Gene Variants With BMIC in Lactating Women

An unadjusted general linear model was applied to study the associations of BMIC and UIC in the subset of lactating women (*n* = 55) with the recessive genetic model for rs4808708_(G/A)_, rs7255301_(G/A)_, rs73520743_(A/C)_, rs112076606_(A/G)_, rs73520745_(G/A)_, rs775249401_(G/A)_; rs34850953_(G/T)_ and rs8103545_(C/T)_ (Table 4). There was a significant difference in UIC between the genotypes of the rs4808708_(G/A)_ variant (*P* = 0.05). Homozygote rs4808708_(GG)_ had higher UIC compared to (A)-allele carriers rs4808708_(AG+AA)_ (*P* = 0.05), whereas BMIC were comparable between the two genotypes (*P* = 0.612) for the same variant. BMIC were different between the rs775249401_(G/A)_ genotypes, whereby the homozygous GG genotype had lower BMIC compared to the (A)-allele carriers rs775249401_(AG+AA)_ (*P* = 0.042). No difference in UIC were apparent for rs775249401_(G/A)_ genotypes. The variant rs112076606_(A/G)_ showed a trend toward a significant association with both BMIC and UIC (*P* = 0.051 and *P* = 0.081). The homozygotes AA genotype had higher median concentrations compared to the G-allele carriers rs112076606_(GA+GG)_.

**Table 4:** Association of SLC5A5 variants with breast milk iodine concentrations and urinary iodine concentrations (*n* = 55).

**Genetic variant**	**(M):(m)**	**MM**	**Mm + mm**	*P*-value^*^
**Breast milk iodine concentration (μg/L)**				
rs4808708	G:A	108.1 (67.3–175.5)	107.9 (46.8–149.7)	0.612
rs7255301	G:A	112.9 (72.5–155.0)	103.7 (44.1–150.1)	0.802
rs73520743	C:A	103.7 (57.7–168.9)	109.2 (83.4–191.1)	0.589
rs112076606	A:G	142.1 (69.4–201.0)	83.3 (39.4–143.4)	0.051
rs73520745	G:A	109.2 (63.0–172.2)	89.1 (68.0–148.4)	0.807
rs775249401	G:A	86.8 (54.9–167.9)	143.9 (122.4–169.3)	0.042
rs34850953	G:T	97.6 (60.5–147.1)	142.1 (41.5–219.4)	0.792
rs8103545	C:T	108.4 (89.1–51.1)	127.1 (89.1–51.1)	0.561
**Urinary Iodine concentration (μg/L)**				
rs4808708	G:A	111.4 (71.2–154.8)	70.1 (45.6–82.8)	0.050
rs7255301	G:A	108.0 (66.4–137.5)	82.4 (60.8–165.1)	0.967
rs73520743	C:A	103.2 (66.2–137.6)	91.1 (65.0–155.1)	0.267
rs112076606	A:G	133.0 (72.2–153.7)	81.4 (58.4–119.1)	0.081
rs73520745	G:A	91.1 (62.2–133.9)	137.7 (69.6–178.3)	0.409
rs775249401	G:A	102.0 (66.4–137.4)	81.1 (48.6–145.2)	0.912
rs34850953	G:T	102.0 (67.2–137.6)	66.1 (47.2–154.0)	0.739
rs8103545	C:T	91.1 (54.7–133.9)	137.7 (66.4–183.1)	0.345

*M, reference allele in this study population; m, alternative allele in this study population*,

* *p-value for the unadjusted general linear model, significance set at p ≤ 0.05*.

**Figure 1:**
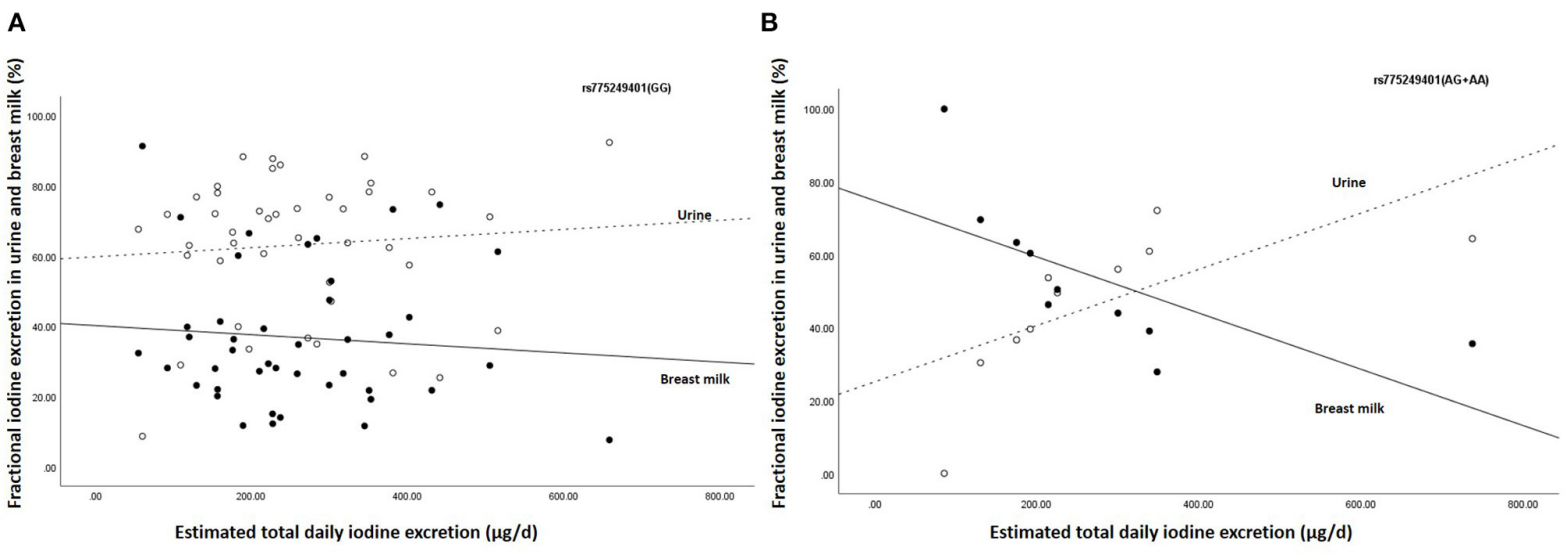
Association between estimated daily iodine excretion and fractional iodine excretion in breast milk and urine for **(A)** the homozygous G group (*n* = 43) and **(B)** the (A) allele carriers (AA + AG) (*n* = 10). The open data points represent fractional excretion of iodine in urine and the filled data points, the fractional excretion in breast milk. The dotted line is the regression line for fractional excretion of iodine in urine. The solid line is the regression line for fractional excretion of iodine in breast milk.

Furthermore, the estimated breast milk iodine excretion for rs775249401_(GG)_ was 67.7 (42.9–131.1) μg/d, while estimated breast milk iodine excretion of the (A)-allele carriers of rs775249401_(AG+AA)_ was 112.2 (95.5–132.1) μg/d. All the (A)-allele carriers (100%) of rs775249401_(AG+AA)_ had a BMIC above 100 μg/L, whereas 39.5% the homozygous GG [rs775249401_(GG)_] had a BMIC ≥100 μg/L. Figure 1 shows the associations between estimated total daily iodine excretion and fractional excretion in urine and breast milk for rs775249401_(G/A)_. The homozygotes GG genotype had a higher fraction of iodine excreted in urine (63.2%) than in breast milk (36.8%) (Figure 1A). The fractional excretion of iodine in urine and breast milk remained constant across the range of estimated total daily iodine excretion. In the (A)-allele carriers rs775249401_(AG+AA)_, fractional iodine excretion in urine and breast milk plotted against total daily iodine excretion (Figure 1B) shows that a higher fraction of iodine was excreted in breast milk than urine when estimated total daily iodine excretion was lower than 350 μg/day, while the fraction of iodine excreted in breast milk was lower than urine above this threshold.

**Figure 2:**
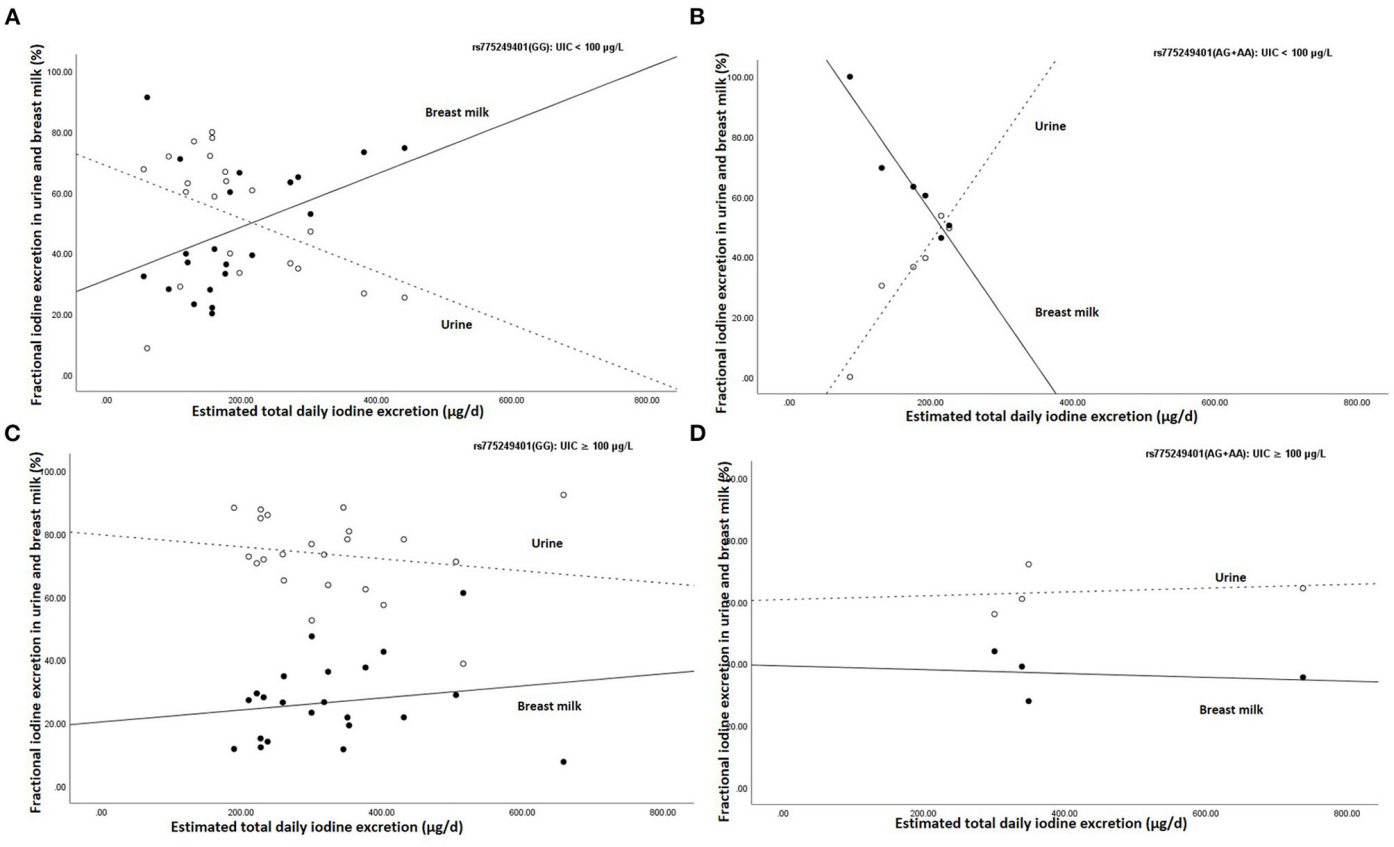
Association between estimated daily iodine and fractional iodine excretion in breast milk and urine **(A)** the homozygous G group (rs775249401_(GG)_) with UIC <100 μg/L (*n* = 21), **(B)** the (A) allele carriers for the rs775249401_(AG+AA)_ with UIC <100 μg/L (*n* = 6), **(C)** the homozygous G group (rs775249401_(GG)_) with UIC ≥100 μg/L (*n* = 22) and **(D)** the (A) allele carriers for the rs775249401_(AG+AA)_ with UIC ≥100 μg/L (*n* = 4). The open data points represent fractional excretion of iodine in urine and the filled data points, the fractional excretion in breast milk. The dotted line is the regression line for fractional excretion of iodine in urine. The solid line is the regression line for fractional excretion of iodine in breast milk.

Figure 2 shows the association between estimated total daily iodine excretion and fractional excretion in urine and breast milk in homozygotes GG (rs775249401_(GG)_) and in the (A)-allele carriers of rs775249401_(AG+AA)_, stratified by UIC (UIC <100 μg/L vs. UIC≥100 μg/L). The fractional excretion of iodine in breast milk for rs775249401_(GG)_ with UIC <100 μg/L was lower than in urine when estimated total daily iodine excretion was low, but higher with higher estimated total daily iodine excretion and exceeded the proportion of iodine excreted in urine (Figure 2A). In contrast, fractional excretion of iodine in breast milk of the(A)-allele carriers with UIC <100 μg/L, was higher than in urine when the estimated total daily iodine excretion was low, but lower with a higher estimated total daily iodine excretion (Figure 2B). In both the rs775249401_(GG)_ and rs775249401_(AG+AA)_ genotype groups with UIC ≥100 μg/L, fractional excretion of iodine in breast milk was lower than fractional excretion of iodine into the urine (Figures 2C,D, respectively) and remained constant across estimated total daily iodine excretion. The association between rs775249401_(G/A)_ and BMIC had a Cohens' d of 0.88 and a partial eta squared η^2^ = 0.078.

## Discussion

To our knowledge, this is the first study to assess the role of genetics in the transfer of iodine from plasma to breast milk in healthy lactating women of African descent using a targeted NGS approach. Genetic variants in the *SLC5A5* gene in women of African descent living in urban South Africa were characterized and studied in the context of iodine transfer from plasma to breast milk during lactation. Our results suggest rs775249401_(G/A)_ to be a candidate variant for mediating the transfer of iodine from plasma into breast milk, specifically among lactating women with poor iodine status. The function of the variant may not be observed when the iodine status is adequate, suggesting that adequate iodine intake may counter the low iodine transfer associated with rs775249401_(GG)_.

We observed that in individuals harboring the rs775249401_(GG)_ genotype fractional excretion of iodine into the breast milk was lower than fractional excretion of iodine into urine with low iodine status, while in the rs775249401_(AG+AA)_ group fractional excretion of iodine into the breast milk was higher even when iodine status was low. Thus, lactating women carrying an (A) allele for rs775249401_(AG+AA)_ (19%; 10/53) had an adaptive advantage to maintain optimal levels of BMIC despite suboptimal iodine status. Our findings suggest a positive genetic drift from the ancestral rs775249401_(GG)_ to the alternative allele to rs775249401_(AG+AA)_, which leads to preferential excretion of iodine into the breast milk when iodine status is low. This preferential excretion of iodine in breast milk rather than in urine is likely a result of an increased expression of the NIS in the lactating breast and further supports the plausibility of a regulatory potential by the rs775249401_(G/A)_.

The rs775249401_(G/A)_ variant is located in a promoter region between exon 4 and 5 (24), which is responsible for transcriptional control. It is speculated that the variant interferes with the affinity of transcription factors (TFs) to bind to their idyllic binding sites (25), thus altering affinity to transcription factors (25). The adaptation observed in our study suggests a physiological benefit for the infant, in that it ensures sufficient iodine in breast milk, especially during periods of low iodine intakes. Furthermore, the rs775249401_(G/A)_ variant is mainly present in African and not in Caucasian populations according to the Genome Aggregation Database (26). The variant rs775249401_(G/A)_ has a minor allele frequency (MAF) of 0.028 in this study population, which is higher than reported in the genome-wide association study for Africa populations (0.015) (24). A total of 14 missense and non-sense mutations in the *SLC5A5* gene have been previously described in association with iodide transport defects in the thyroid (27). However, a better understanding of the impact that genetic variations in the *SLC5A5* gene have on BMIC is as important.

Out of the 10 variants explored in our study, only rs775249401 was significantly associated with BMIC. One other variant, rs4808708_(G/A)_, was associated with UIC but not BMIC. The variant rs112076606_(A/G)_ showed a trend toward significance with both BMIC and UIC, whereby participants with the genotype rs112076606_(AA)_ had a higher BMIC and UIC compared to rs112076606 _(AG+GG)_ carriers, suggesting AA homozygotes to have a higher overall iodine excretion compared to their G allele carrier counterparts.

To our knowledge, this is the first study to assess the role of genetics in the transfer of iodine from plasma to breast milk in healthy lactating women. Furthermore, our participants were healthy participants with no known history of thyroid disease. However, a major limitation of this study is the small sample size; based on an *a posteriori* sample size calculation, our study was only powered to determine associations of medium to large effect sizes. Thus, exploring the relationship of variants in the SLC5A5 gene locus in other studies with larger sample sizes is highly recommended.

## Conclusion

Our results indicate that genetics may play an important role in the transfer of iodine into breast milk. The *SLC5A5* gene variant rs775249401_(G/A)_ seems to be a candidate variant for further investigation. The A-allele carriers of rs775249401_(AG+AA)_ are likely to have higher iodine transfer into breast milk when in an iodine-deficient state compared to the homozygous GG group. Our results suggest that genetic variations in the SLC5A5 gene may play an important role in the transfer of iodine from plasma into breast milk and may partially explain variability in BMIC independent of maternal iodine intake. Therefore, these findings could contribute toward the body of evidence to improve precision nutrition strategies.

## Data Availability Statement

The data generated for the study are deposited in the sequence read archive (SRA), bioproject number (PRJNA735618), https://www.ncbi.nlm.nih.gov/bioproject/PRJNA735618.

## Ethics Statement

This study was conducted according to the guidelines laid down in the Declaration of Helsinki, and all procedures involving research study participants were approved by the Human Research Ethics Committee of the North-West University and the University of the Witwatersrand, Johannesburg. Permission to perform the NuPED study was given by the CEO of RMMCH, the RMMCH research review committee, the Gauteng Department of Health and the Johannesburg Health District's District Research Committee. Written informed consent was obtained from all participants before enrolment. Consent for genetic testing were obtained.

## Author Contributions

LZ, JB, JN, and SS conceptualized and designed the study and wrote the first draft of the manuscript. SS, JB, LZ, EAS, LM, and CMS executed the study and collected data. LZ, MS, JB, and SS performed biochemical and statistical analyses. All authors critically evaluate the manuscript.

## Conflict of Interest

The authors declare that the research was conducted in the absence of any commercial or financial relationships that could be construed as a potential conflict of interest.

## Publisher's Note

All claims expressed in this article are solely those of the authors and do not necessarily represent those of their affiliated organizations, or those of the publisher, the editors and the reviewers. Any product that may be evaluated in this article, or claim that may be made by its manufacturer, is not guaranteed or endorsed by the publisher.
